# Valosin-Containing Protein Contributes to Plexiform Neurofibroma Formation and Represents a Novel Therapeutic Target

**DOI:** 10.3390/cells15090848

**Published:** 2026-05-06

**Authors:** Lalitha Gopalan, Youjin Na, Liang Hu, Ashley Hall, Mi-Ok Kim, Eva Dombi, Sara Szabo, Nancy Ratner, Gang Huang, Jianqiang Wu

**Affiliations:** 1 Division of Experimental Hematology and Cancer Biology, Cancer & Blood Diseases Institute, Cincinnati Children’s Hospital Medical Center, 3333 Burnet Ave., Cincinnati, OH 45229, USA; lalithagopalan7@gmail.com (L.G.); na.152@osu.edu (Y.N.); liang.hu@cchmc.org (L.H.); ashley.mahler@cchmc.org (A.H.); nancy.ratner@cchmc.org (N.R.); 2 Department of Epidemiology and Biostatistics, University of California San Francisco, 1450 3rd Street, San Francisco, CA 94143, USA; miok.kim@ucsf.edu; 3 Pediatric Oncology Branch, National Cancer Institute, Bethesda, MD 20892, USA; dombie@mail.nih.gov; 4 Department of Pediatrics, Cincinnati Children’s Hospital Medical Center, University of Cincinnati, Cincinnati, OH 45229, USA; sara.szabo@cchmc.org; 5 Department of Pediatric Pathology, Cincinnati Children’s Hospital Medical Center, University of Cincinnati, Cincinnati, OH 45267, USA; 6 Department of Pediatrics, University of Cincinnati College of Medicine, Cincinnati, OH 45267, USA; 7 Department of Cell Systems and Anatomy, Joe R. and Teresa Lozano Long School of Medicine, UT Health San Antonio, San Antonio, TX 78229, USA; huangg1@uthscsa.edu

**Keywords:** neurofibromin, plexiform neurofibroma, Schwann cell, p97/VCP, CB-5083, PD0325901

## Abstract

**Highlights:**

**What are the main findings?**

- VCP expression is increased in both mouse and human plexiform neurofibromas (PNFs).
- VCP contributes to neurofibroma formation, and targeted inhibition of VCP reduces mouse PNF growth.

**What are the implications of the main findings?**

- VCP plays a pro-tumorigenic role in PNF formation, extending its known roles beyond regulating proteostasis and multiple cellular processes.
- Inhibition of VCP effectively reduces PNF volume, suggesting that targeting VCP may represent a novel therapeutic strategy for patients with PNFs.

**Abstract:**

Neurofibromatosis type 1 (NF1) patients are predisposed to develop plexiform neurofibromas (PNFs). By cross-comparison of RNA sequencing and RUNX1-CHIP sequencing data on mouse PNFs, we found that transcripts encoding the NF1-interacting p97/valosin-containing protein (VCP) gene are overexpressed in PNFs. Co-immunoprecipitation confirmed that VCP bounded to neurofibromin. Western blot and immunostaining confirmed VCP overexpression in both mouse and human PNFs. Treatment of primary mouse PNF Schwann cells with CB-5083, a p97/VCP inhibitor, led to accumulation of poly-ubiquitinated proteins and generation of irresolvable proteotoxic stress. Pharmacological or genetic inhibition of VCP reduced mouse PNF cell-derived sphere number, and genetic inhibition of *Vcp* in Schwann cell precursors decreased tumor-like lesion numbers in a cell transplantation model. In vivo treatment with CB-5083 in *Nf1^fl/fl^;DhhCre* PNF mice significantly inhibited cell proliferation, increased cell apoptosis and reduced PNF volume. The combination with a MEK inhibitor did not increase efficacy compared to the single agent, supporting the hypothesis that VCP functions in parallel to, and may be modulated by, RAS–MAPK signaling under stress or oncogenic conditions. The significant effects of VCP inhibition in this pre-clinical study suggest a potential novel therapy for patients with PNFs.

## 1. Introduction

Neurofibromatosis type 1 (NF1) is classified as rare, but of the rare diseases is relatively common, affecting 1 in 2500 people worldwide [1,2,3]. One of the hallmarks is that about 30–50% of NF1 patients develop plexiform neurofibromas (PNFs), benign Schwann cell (SC) tumors. PNFs can transform into malignant peripheral nerve sheath tumors, lethal sarcomas [4,5]. PNFs can be large and locally invasive and can cause substantial morbidity—including pain, neurologic deficit, motor dysfunction, disfiguration, and even death. The mainstay of PNF therapy, surgery, is sometimes impossible due to tumor integration into nerves in critical anatomic sites. There are two FDA-approved MEK inhibitors (MEKis), selumetinib and mirdametinib, for the treatment of PNFs. These two inhibitors shrink PNFs in 40–70% of NF1 patients, but tumor shrinkage is modest in most tumors, and some tumors regrow when the drug is withdrawn [6]. In addition, patients receiving MEKi treatment often experience side effects—including elevated creatine phosphokinase, acneiform rash, and paronychia [6]. Finally, given that PNF onset is during early childhood [7], taking selumetinib or mirdametinib for life can be problematic due to adherence and side effect issues. Therefore, new effective and durable therapeutic strategies are urgently needed.

Valosin-containing protein (VCP, also called p97) is a homohexamer consisting of an N-terminal domain, two ATPase (D1 and D2) domains, a flexible linker region, and a C-terminal domain. VCP is an AAA+ ATPase that, by interaction with a wide variety of partners and cofactors, regulates proteostasis and multiple other cellular processes (e.g., lysosomal dynamic stability) [8,9,10]. VCP, usually through its ATPase activity, regulates ubiquitination [10,11]. VCP also regulates autophagy [12,13]. In neurons, VCP interacts directly with neurofibromin, the *NF1* gene-encoding protein [14], and heterozygous *Nf1* (*Nf1^+/−^*) neurons regulate protein synthesis in the endoplasmic reticulum (ER) via VCP [12]. In addition to regulating proteostasis, VCP also suppresses apoptosis by an unfolded protein response in normal and tumor cells [15,16,17,18,19]. Importantly, in many human tumors, VCP mRNA and proteins are elevated, and multiple in vivo studies strongly suggest that VCP promotes tumor growth [10,20,21].

To develop effective and durable therapies for PNF, it is critical to understand the signaling pathways controlling PNF formation and growth—particularly the MEK-independent and MEK synergistic pathways. In this study, we showed that VCP interacts with neurofibromin in SCs, and its protein is overexpressed in both mouse and human PNFs. Pharmacological or genetic inhibition of VCP reduced mouse neurofibroma sphere number, and genetic inhibition of *Vcp* in Schwann cell precursors (SCPs) decreased tumor-like lesion numbers in a cell transplantation model. In vivo treatment with CB-5083 in *Nf1^fl/fl^;DhhCre* PNF mice significantly reduced PNF volume, suggesting that targeting VCP might be an alternative therapeutic strategy for patients with PNFs.

## 2. Materials and Methods

### 2.1. Animals

All animal procedures were approved by the animal care and use committees of Cincinnati Children’s Hospital Medical Center. We followed Institutional Animal Care and Use Committee (IACUC) guidelines for animal subjects. We housed mice in temperature- and humidity-controlled facilities with free access to food and water and a 12 h light–dark cycle. Floxed −*Nf1* mice (*Nf1^fl/fl^*) have been described previously [22] and were on a mixed 129/BL/6 background. *DhhCre* transgenic mice contain an inserted nuclear-localized *Cre* recombinase gene that is regulated by mouse desert hedgehog (*Dhh*) promoter/regulatory regions and were maintained on a C57/BL/6 background [23]. We bred *DhhCre* mice onto *Nf1^fl/fl^* mice to obtain the F1 generation (*Nf1^fl/+^*;*DhhCre*^+^); we bred male F1 mice with female *Nf1^fl/fl^* mice to obtain *Nf1^fl/fl^;DhhCre* mice. Genotyping was performed as described previously [24].

### 2.2. Mouse MRI and Volumetric Measurements

We performed mouse MRI and volumetric measurements as described previously [25]. Specifically, we scanned *Nf1^fl/fl^;DhhCre* PNF-bearing mice by MRI at 5, 7 and 9 months of age to monitor PNFs using the 7 Tesla Bruker Biospec system (Billerica, MA, USA) equipped with 400 G/cm gradients in the Imaging Resource Center at Cincinnati Children’s Hospital. We determined tumor volume changes using MRI-based volumetric measurements as described previously [26]. We reported all volumes as combined tumor volume in individual mice. The person who performed the volumetric measurements was blind to the mouse treatment groups.

### 2.3. Reagents

CB-5083 (ChemGood, Henrico, VA, USA) and PD0325901 (mirdametinib, Selleck Chemicals, Houston, TX, USA) were dissolved in 0.5% [*w*/*v*] methylcellulose (Neta Scientific, Hainesport, NJ, USA; #SIAL-M0262) solution with 0.2% [*v*/*v*] polysorbate 80 [Tween 80] (Fisher Scientific, Waltham, MA, USA; # BP338-500) for mouse dosing. CB-5083 was sonicated at medium-level energy for 2 min on ice before use.

### 2.4. Immunoprecipitation Assay

Embryonic day 12.5 WT mouse SCs (1 × 10^7^) were lysed in cold lysis buffer containing 50 mM Tris-HCl (pH 7.5), 150 mM NaCl, 1% NP-40, and 1× protease inhibitors (VWR, Radnor, PA, USA; #PIA32953), followed by incubation on ice for 30 min. The lysate was then centrifuged at 12,000× *g* for 10 min at 4 °C, and the supernatant was collected. To reduce nonspecific binding, 20 µL of protein A/G plus-agarose beads (Santa Cruz Biotechnology, Dallas, TX, USA; #sc-2003) were added to 750 µg of total protein lysate and incubated at 4 °C for 30 min with rotation. The mixture was centrifuged at 2000× *g* for 1 min at 4 °C, and the supernatant was transferred to a new tube. Five micrograms of neurofibromin antibody (Santa Cruz Biotechnology; #sc-398267) or normal mouse IgG 1κ control (Santa Cruz Biotechnology; #sc-2025) were added and incubated overnight at 4 °C. The following day, 30 µL of pre-washed protein A/G agarose beads were added and incubated for 2 h at 4 °C with rotation. The beads were then pelleted by centrifugation and washed three times with cold PBS wash buffer containing 300 mM NaCl and 0.2%NP-40. Subsequently, 50 µL of SDS sample buffer was added, and samples were heated at 95 °C for 5 min. The resulting immunoprecipitated complexes were separated by SDS-PAGE and transferred to PVDF membranes for immunoblotting. Detection was performed using anti-NF1 (Cell Signaling Technology, Danver, MA, USA; #14623, 1:1000) and VCP (Proteintech, Rosemont, IL, USA; #10736-1-AP, 1:2000) antibodies.

### 2.5. Western Blots

Mouse spheres, PNFs or WT mouse dorsal root ganglia (DRG)/nerve lysates were used for Western blots as described previously [27]. For immunoblotting, we used the following antibodies: VCP (Proteintech; # 10736-1-AP, 1:2000); pERK (Cell Signaling Technology; #4370, 1:2000), ERK (Cell Signaling Technology; #4695, 1:2000), Ubiquitin (Cell Signaling Technology; #58395, 1:1000), CHOP (Cell Signaling Technology; #5554, 1:1000), GAPDH (Cell Signaling Technology; #3683, 1:3000), or β-actin (Cell Signaling Technology; #5125, 1:5000). Antibody binding to the membrane was visualized using a chemiluminescent detection system (EMD Millipore, Burlington, MA, USA; #WBKLS0500).

### 2.6. Generation of shRNAs and Lentiviral Transduction

We purchased two VCP-specific shRNA-expressing lentiviral plasmids (TRCN0000008410 [#10] and TRCN0000008413 [#11]) from Sigma-Aldrich (St. Louis, MO, USA) and produced lentivirus particles by transient co-transfection of 293T cells as described previously [28]. We concentrated the lentiviral particles using a commercial Lenti-X concentrator (Takara Bio Inc., Shiga, Japan; # 631231). We determined the titer of lentivirus using the Lenti-X™ GoStix™ Plus Kit (Takara; #631280) in combination with an iPhone with the GoStix Plus app installed. Briefly, 20 µL of virus was applied to a GoStix cassette and incubated for 10 min to allow the development of bands corresponding to lentiviral p24 (control) and the test virus. Bands were scanned using an iPhone, and lentiviral titers were calculated by comparing band intensities using the GoStix Plus app (Version 1.2.7).

### 2.7. Mouse PNF Cell-Derived Sphere Culture and Treatment

We cultured mouse PNF cell-derived spheres. Briefly, we cut PNFs into around 1 mm^3^ pieces and dissociated them in 20 mL of L-15 (Mediatech, Herndon, VA, USA; #10-045-CV) containing 0.5 mg/mL of collagenase type 1 (Worthington Biochemical, Lakewood, NJ, USA; #LS004196) and 2.5 mg/mL of Dispase protease type II (Roche, Indianapolis, IN, USA; #C756V28) at 37 °C for 3 h with 200 rpm shaking. We plated trypan-blue-negative single cells at 4 × 10^4^ cells per well in 24-well low-binding plates (Fisher Scientific, # Corning 3473) with 1 mL of medium containing DMEM:F-12 (3:1), 20 ng/mL of rhEGF (R&D Systems, Minneapolis, MN, USA; #236-EG-200), 20 ng/mL rh bFGF (PeproTech, Cranbury, NJ, USA; #450-33-50 µg), 1% B-27 supplement (Life Technologies, Carlsbad, CA, USA; #17504-044), and 2 mg/mL of heparin (Sigma-Aldrich; #H3149-10KU). Spheres were cultured at 37 °C at 5% CO_2_. We used secondary spheres for each experiment.

For treatment, we seeded dissociated live cells from primary spheres into 24-well ultra-low attachment plates at a density of 4 × 10^4^ cells/well for 48–72 h and then treated them with vehicle, CB-5083, sh*Vcp* or sh*NT* lentivirus. For drug treatment, CB-5083 at 0 nM, 1 nM, 10 nM, 100 nM, 1000 nM, and 10,000 nM were used. After 4 days, we counted the sphere numbers using a phase-contrast microscope (Olympus CKX41, Tokyo, Japan) and collected them for Western blotting. For lentivirus treatment, we transduced secondary PNF cell-derived spheres with purified sh*Vcp* or sh*NT* (Sigma-Aldrich) at the multiplicity of infection of 1:10 and collected spheres at 3 days for Western blotting, and at 4 days for sphere number counting.

### 2.8. Tumorigenesis Assay in NSG Mice

We treated *Nf1^fl/fl^;DhhCre* mouse PNF cell-derived secondary spheres with sh*NT* or *shVcp (#11)* lentivirus at multiplicity of infection of 1:10 for 3 days and dissociated them into single cells. We subcutaneously injected 5 × 10^5^ dissociated sphere cells/injection with 33% Matrigel (Fisher Scientific; #CB-40234) into the right flanks of NSG mice (males and females, Harlan, Indianapolis, IN, USA) as described previously [27]. After 3 months, we dissected lesions under a dissection microscope to confirm visible tumors and counted tumor number. Tumors were processed for H&E staining.

### 2.9. Quantitative Real-Time PCR (qRT-PCR)

We isolated total RNA from mouse tissues using the Qiagen RNeasy Kit (Hilden, Germany). We synthesized the cDNA using the High-Capacity cDNA Reverse Transcription Kit (Thermo Scientific, Waltham, MA, USA; #4368814). We performed qRT-PCR using SYBR Green (Applied Biosystems, Foster City, CA, USA; #4368708). We calculated the relative RNA expression change using the ΔΔCt method. Data represented three independent experiments with triplicates of each sample. We used the following mouse primers: *Svip:* Forward: 5′-GCTTCCAGACTTAGTGCCTTTA-3′; reverse; 5′-CGTCCTACGGACATGTAACTT-3′. Tublin: Forward: 5′-CATTCAGGGCTCCATCAAAT-3′; reverse: 5′-GCCCTACAACTCCATCCTCA-3′.

### 2.10. ATPase Assay

ATPase assay was performed according to Abcam ATPase Assay instructions (colorimetric assay). Briefly, CB-5083-treated cells were rapidly homogenized in 400 µL of ice-cold ATPase assay buffer, incubated on ice for 10 min, and centrifuged at 10,000× *g* for 10 min at 4 °C. For the assay, duplicate sample aliquots (2 µL) were plated in a clear 96-well plate and adjusted to a final volume of 100 µL with buffer. A fresh phosphate standard curve (0 to 5 nmol/well) and appropriate controls (reagent and positive) were prepared in parallel using the ATPase assay kit (Abcam, Cambridge, MA, USA; #ab234055). After initiating the enzymatic reaction by adding ATP (ATP IV) to the positive control, reagent control and test samples, they were read using an ATPase assay developer, followed by a 30 min incubation at 25 °C. The optical density (OD) was measured at 650 nm using a microplate reader. Raw absorbance values were background-corrected and interpolated against the phosphate standard curve to determine the amount of phosphate generated (nmol). Finally, sample ATPase activity was calculated using the following equation:

$$Sample\;ATPase\;Activity\;(U/mL)=\frac{B}{t\times V}\times D$$

where B is the phosphate generated (nmol), t is the reaction time (30 min), V is the sample volume added to the reaction well (μL), and D is the sample dilution factor. One unit (U) of ATPase is defined as the amount of enzyme required to generate 1.0 µmol of phosphate per minute at pH 7.5 at 25 °C. Each experiment was repeated three times with triplicate samples.

### 2.11. Immunohistochemistry and Immunofluorescence

We sectioned paraformaldehyde-fixed, paraffin-embedded tissue blocks at 10 μm. We processed sections by deparaffinizing in xylene, rehydrating through serial concentrations of ethanol, and then boiling in citrate buffer (pH 6.0) for antigen retrieval. We permeabilized tissues with 0.2% triton-X100/PBS (TBST) for 30 min and washed with PBS three times. After blocking with 10% normal goat serum (Jackson ImmunoResearch, West Grove, PA, USA; #005-000-121) in TBST, we incubated sections with primary antibodies—Ki67 (Cell Signaling Technology; # 12202S, 1:200), VCP (Proteintech; # 10736-1-AP, 1:200), and SVIP (Abcam, Cambridge, MA, USA; #ab122590, 1:250)—overnight at 4 °C and then treated them with biotinylated or fluorescence-dye-conjugated secondary antibodies for 1 h. We detected the biotinylated antibodies with horseradish peroxidase-conjugated streptavidin (Elite ABC, Vector Laboratories, Burlingame, CA, USA; #PK-6100), and stained cell nuclei with hematoxylin. For fluorescence staining, we stained sections with 0.1 μg/mL DAPI (Fisher Scientific; #62248) for nuclear counting and then mounted them in Fluoromount G slide mounting medium (Electron Microscopy Sciences, Hatfield, PA, USA; 17984-25). Images were captured on a Nikon C2 confocal microscope (Nikon Inc./Nikon Americas, Melville, NY, USA).

### 2.12. TUNEL Assay

We performed a TUNEL assay according to the manufacturer’s instructions using the Roche In Situ Cell Death Detection Kit (TMR red, Roche Diagnostics Gmbh, Mannheim, Germany; Ref#12156792910) on deparaffinized sections. Nuclei were stained with DAPI. TUNEL^+^ cells and DAPI-stained nuclei were counted in at least three cross-sections per sample. Data is presented as an average percentage of TUNEL^+^ cells per sample.

### 2.13. Statistical Analysis

We used unpaired 2-tailed Student’s *t*-tests to analyze the significance of cell population percentages and relative mRNA expression when two samples were compared. We used one-way ANOVA to analyze the significance of cell proliferation, apoptosis and sphere numbers per well. Data is reported as the mean ± SD of at least three independent experiments or samples. *p* < 0.05 was considered significant. We used a mixed-effects model to analyze PNF growth. *p*-values were generated with a random-effects model analysis on log-transformed tumor volume data using the SAS (9.4M7) mixed procedure [25]. For the in vivo cell transplantation experiment, we used Fisher’s exact test for *p*-values.

## 3. Results

### 3.1. VCP Interacts with Neurofibromin in SCs and Is Overexpressed in Mouse and Human PNFs

**Figure 1:**
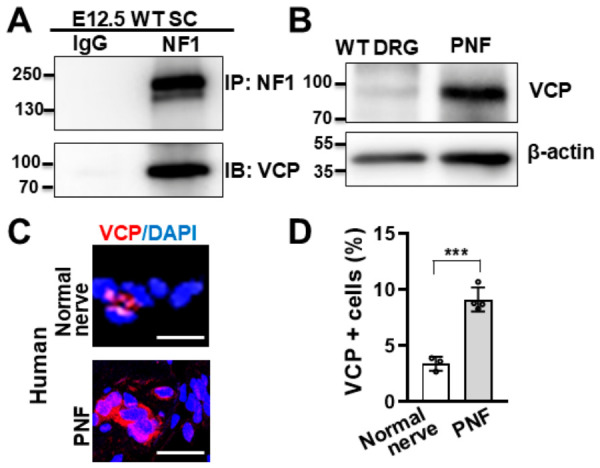
**VCP interacts with neurofibromin in Schwann cells and is overexpressed in mouse and human PNFs.** (**A**) Interaction between neurofibromin and VCP in wild-type (WT) mouse Schwann cells, showing that VCP interacts with neurofibromin. IP: Neurofibromin. Immunoblot: VCP. Control: IgG. (**B**) Western blot of 7-month-old *Nf1^fl/fl^;DhhCre* mouse PNFs and age-matched WT mouse DRG, showing VCP expression is increased in PNFs. Loading control: β-actin. (**C**) Representative image of immunofluorescence staining of VCP (red) on normal primary human nerve (top) and human PNF (bottom). DAPI used to stain nuclei. Bar: 20 µm. Note: The three normal primary human nerves were: a. M, 18y, anterior vagus; b. M, 15y, nerve; c. M, 18y, ilioinguinal. (**D**) Quantification of percentage of VCP^+^ cells in normal human nerves (left, white bar, *n* = 3) and human PNFs (right, gray bar, *n* = 4) showing significant increase in PNFs compared to normal human nerves. *** *p* < 0.0001.

We previously showed that ER stress protein homoeostasis is unbalanced in PNF SCs/Schwann cell precursors (SCPs) [27]. Given the increased protein synthesis and activation of ER stress pathways observed in PNF cells, we decided to examine upstream components of the ER proteostasis machinery. By comparing PNF RNA sequencing and PNF SC RUNX1-CHIP sequencing data on mouse PNFs, we found that transcripts encoding the NF1-interacting p97/VCP gene were overexpressed in PNFs. To determine if VCP interacts with neurofibromin in SCs, we performed immunoprecipitation on mouse WT SCs. Western blotting confirmed that anti-NF1 immunoprecipates contained VCP, but the protein was not present in the IgG control (Figure 1A), indicating that VCP specifically interacts with neurofibromin in SCs. To check if VCP expression is altered upon loss of *Nf1*, we performed western blots on PNFs from 7-month-old *Nf1^fl/fl^;DhhCre* mice and age-matched WT DRG. We detected increased expression of VCP in PNFs compared to WT mouse DRG (Figure 1B). Immunofluorescence staining on human PNFs showed that there was significantly increased VCP expression in these cells compared to normal human nerve controls (Figure 1C,D). This data suggests that VCP is present in SCs.

### 3.2. RUNX1 Binding to SVIP Indirectly Regulates VCP Expression

**Figure 2:**
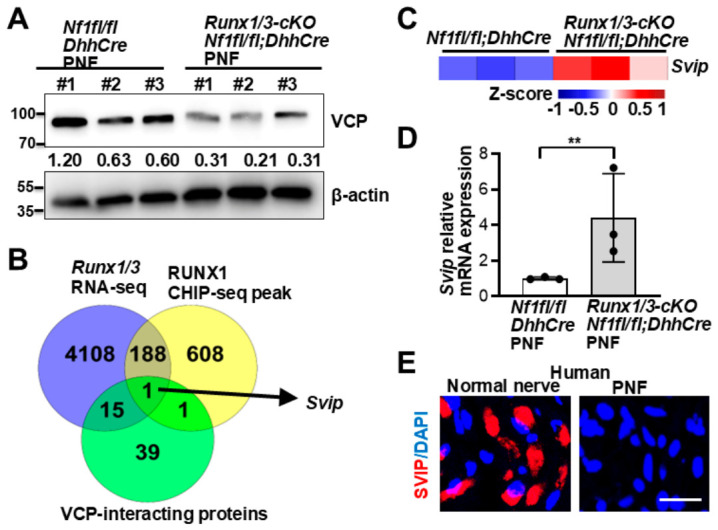
**RUNX1 binding to SVIP indirectly regulates VCP expression.** (**A**) Western blot of VCP in *Nf1^fl/fl^;DhhCre* (*n* = 3) and *Runx1^fl/fl^;Runx3^fl/fl^;Nf1^fl/fl^;DhhCre* (*Rnux1/3-cKO;Nf1^fl/fl^;DhhCre)* (=3) mouse PNFs. Loading control, β-actin. Numbers between the bands indicate the relative intensity of VCP normalized to β-actin. (**B**) Venn diagram of shared gene expression across 3 data sets (*Runx1/3* RNA-seq, RUNX1 CHIP-seq peak, and VCP interaction proteins). (**C**,**D**) Heatmap (**C**) and qRT-PCR (**D**) of *Svip* expression in PNFs from *Nf1^fl/fl^;DhhCre* and *Runx1/3-cKO;Nf1-cKO* mice. *n* = 3 mice/group. (**E**) Representative image of SVIP immunofluorescence (red) in human PNFs (right) vs. normal nerves (left). DAPI stains nuclei. Bar: 20 µM. Note: The three normal primary human nerves were from a. M, 18y, anterior vagus; b. M, 15y, nerve; c. M, 18y, ilioinguinal. **, *p* < 0.01.

We decided to determine how VCP expression is increased in PNFs. We previously showed that RUNX1 contributes to PNF formation [27]. Western blots of VCP on *Nf1^fl/fl^;DhhCre* and *Runx1^fl/fl^;Runx3^fl/fl^;Nf1^fl/fl^;DhhCre (Runx1/3-cKO;Nf1^fl/fl^;DhhCre)* mouse PNFs showed decreased VCP expression in *Runx1/3-cKO; Nf1^fl/fl^;DhhCre* (Figure 2A). Because RUNX1 has not been noted to interact with VCP, we explored potential downstream targets of RUNX1 that might interact with VCP by integrating three published data sets: *Runx1/3-cKO; Nf1^fl/fl^;DhhCre* RNAseq differentially expressed genes (both up and down-regulated genes, 2-fold change, *p* < 0.05) [27]; RUNX1 ChIP-seq peaks located within 3 kb upstream or downstream of transcription start sites [27]; and reported VCP-interacting proteins [9,29,30]. The small VCP-interacting protein (*Svip*) was the only gene common to all three data sets (Figure 2B). This suggests that loss of NF1 increases RUNX1 expression, which in turn promotes RUNX1 binding to SVIP, thereby indirectly regulating VCP expression. In fact, *Svip* mRNA expression was significantly increased in *Runx1/3-cKO; Nf1^fl^^/fl^;DhhCre* PNFs (vs. *Nf1^fl/fl^;DhhCre*) (Figure 2C), and increased relative mRNA expression data was validated by qRT-PCR (Figure 2D). We were unable to identify an anti-SVIP antibody for immunofluorescence staining in mouse PNF. Therefore, we checked SVIP expression levels in human PNFs. Consistent with the anti-correlated expression of SVIP and VCP [8], immunofluorescence staining on human PNFs showed decreased SVIP protein expression compared with normal human nerve controls (Figure 2E).

### 3.3. Overwhelming Proteotoxic Stress by Targeting VCP Inhibits Cell Growth and Induces Protein Ubiquitination in PNF SCs

**Figure 3:**
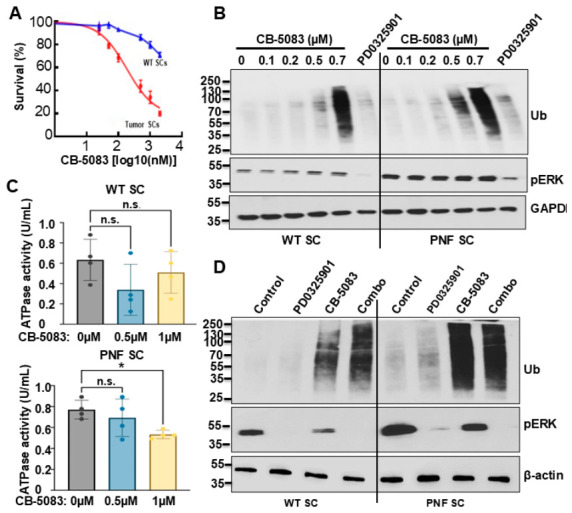
**Overwhelming proteotoxic stress by targeting VCP inhibits cell growth and induces ubiquitination in PNF SCs.** (**A**) MTS assay showing that inhibition of VCP differentially inhibits WT (blue) and PNF (red) Schwann cell growth. (**B**) Western blots showing dose response and differential ubiquitination effects following 6 h treatment with CB-5083 on mouse Schwann cells derived from WT DRG or PNFs. PD0325901 was used for comparison. Loading control, GAPDH. (**C**) Quantification of ATPase activity in CB-5083–treated WT or PNF Schwann cells for 6 h showing 1 µM CB-5083 treatment significantly reduces PNF Schwann cell ATPase activity (n = 4/group). * *p* < 0.05, n.s., no significant difference. (**D**) Western blots showing protein ubiquitination effects or p-ERK activity change following 6 h treatment with CB-5083 (0.5 µM) and PD0325901 (1 µM) alone or in combination on mouse Schwann cells derived from WT DRG or PNFs. Combo = 0.5 µM CB-5083 + PD0325901 1 µM. Loading control, β-actin.

Since the anti-tumor activity of MEK inhibitors (PD0325901 or selumetinib) is not cytotoxic and is not durable post-treatment [6,31], we tested whether targeting VCP is cytotoxic for NF1 PNF cells. The D2 domain of VCP is the primary driver of ATPase activity and conformational changes, and CB-5083 is a selective VCP D2 domain inhibitor [32]. CB-5083 treatment led to a dramatic dose-dependent growth inhibition in PNF-derived SCs compared to WT SCs; importantly, the cytotoxic effect on WT SCs was relatively modest (Figure 3A). Similarly, protein ubiquitination increased in CB-5083-treated PNF-derived SCs 6 h after treatment and was also dose-dependent (Figure 3B); at 500 nM the difference in protein ubiquitination between WT and PNF cells was most dramatic, suggesting a potential therapeutic dose window. Interestingly, CB-5083 did not change *p*-ERK expression (an MAPK inhibition readout) even at 700 nM, suggesting that VCP might be MEK/ERK pathway-independent or regulated by the MEK/ERK pathway. CB-5083 is a selective inhibitor of VCP. VCP requires ATP hydrolysis to extract and process protein ubiquitination. Therefore, inhibition of VCP by CB-5083 is expected to impair ATP hydrolysis, reducing overall ATPase activity. To determine if that is the case here, we performed total ATPase activity assays on CB-5083 treated mouse WT and PNF SCs. ATPase activity inhibition was similar in both 0.5 µm and 1 µM CB-5083-treated WT SCs. On the contrary, ATPase activity was significantly reduced in 1 µM CB-5083-treated PNF SCs compared to vehicle control (Figure 3C). The reduction in ATPase activity following CB-5083 treatment is consistent with on-target inhibition of VCP, suggesting that VCP contributes to the overall cellular ATPase activity measured in these two cell types.

We next tested if VCPi + MEKi combination treatment leads to additive effects. We treated WT and PNF SCs with control, PD0325901 (1 µM), CB-5083 (0.5 µM), or the combination for 6 h. Western blot analysis showed that the combination treatment inhibited p-ERK expression and induced protein ubiquitination simultaneously. The MEKi reduced p-ERK expression, whereas CB-5083 induced protein ubiquitination in both WT and PNF SCs. Consistently, CB-5083 +/− PD0325901 showed stronger effects on protein ubiquitination in PNF SCs vs. WT SCs (Figure 3D).

### 3.4. Pharmacological and Genetic Inhibition of VCP Decreases Mouse PNF Cell-Derived Sphere Number In Vitro

**Figure 4:**
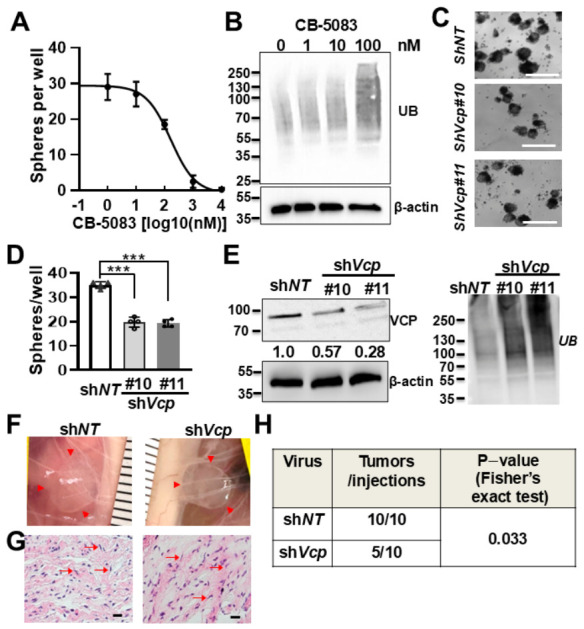
**Pharmacological and genetic inhibition of VCP decreases mouse Schwann cell precursor growth in vitro and tumorigenesis in vivo.** (**A**) Dose–response curve showing decreased numbers of mouse neurofibroma-derived spheres during CB-5083 treatment for 4 days. Three independent experiments were performed. (**B**) Western blot showing protein ubiquitination on spheres from A. Loading control: β-actin. (**C**) Representative images of mouse PNF cell-derived spheres treated with sh*NT* (top), sh*Vcp#10* (middle), and sh*Vcp#11* (bottom). Bar = 100 µm. (**D**) Quantification showing mouse neurofibroma-derived sphere numbers were significantly reduced in spheres treated with two sh*Vcp*-expressing lentivirus (sh*Vcp#10* and sh*Vcp#11*) compared to sh*NT* after 4 days. Three independent PNFs were used. Each experiment was performed in triplicate. ***, *p* < 0.001. (**E**) Western blot showing efficiently inhibited VCP expression by sh*Vcp* (left) and increased protein ubiquitination (right) in mouse PNF cell-derived spheres. Numbers between the bands indicate VCP intensity normalized to β-actin and expressed relative to the sh*NT* control. (**F**) Representative images of tumor-like lesions under reflected skin in NSG mice injected with sh*NT* (left) or sh*Vcp#11* (right)-treated PNF cell-derived sphere cells. Red arrowheads point to tumor-like lesions. Ruler shows 1 mm markings. (**G**) H&E-stained sections of tumors from (**F**) showing spindle cells (red arrows). (**H**) Decreased tumor-like lesion formation rate by transplantation of sh*Vcp* lentivirus-treated spheres compared to that of sh*NT* control-treated sphere. Fisher’s exact test was performed.

SCPs are the cell of origin for PNFs. To test if inhibition of VCP affects SCP growth and/or tumorigenesis, we used a neurofibroma sphere culture system, in which SCPs grow as self-renewing spheres that can be passaged in vitro for 2–3 passages. CB-5083 inhibited sphere formation (numbers) in a dose-dependent manner (Figure 4A). Exposure (4 days) to CB-5083 at 100 nM led to accumulation of protein ubiquitination (Figure 4B), which was not observed after short-term (6 h) treatment at this dose (Figure 3B). To verify that this inhibitory effect was specifically caused by VCP, we treated *Nf1^fl/fl^;DhhCre* mouse PNF SC-derived secondary spheres with two different *Vcp* sh-RNAs (sh*Vcp#10* and sh*Vcp#11*) for 4 days. Each sh*Vcp* significantly decreased visible sphere numbers compared to the sh*NT* control (Figure 4C,D). VCP knockdown was confirmed by Western blotting (Figure 4D, left). Consistently, VCP knockdown increased protein ubiquitination (Figure 4D, right). These results indicate that VCP is important for SCP growth.

### 3.5. VCP Contributes to Neurofibroma Initiation

To test whether VCP reduction in neurofibroma sphere numbers decreases tumorigenic potential in vivo, we dissociated 3-day sh*Vcp#11* or sh*NT* lentivirus-treated *Nf1^fl/fl^;DhhCre* mouse PNF-derived secondary tumor sphere cells into single cells and injected them subcutaneously into NSG mice. Three months after transplantation, all mice (10 of 10) grafted with sh*NT*-treated spheres showed neurofibroma-like micro-lesions, while only 5 of 10 NSG mice transplanted with sh*Vcp*-treated sphere cells showed detectable lesions (*p* < 0.05) (Figure 4F,G). H&E staining showed features of neurofibroma lesions including spindle-shaped cells and collagen (red) in both the sh*NT* and sh*Vcp* groups, but no difference was observed (Figure 4H). Thus, VCP loss compromised neurofibroma initiation in this assay. Global *Vcp*-knockout mice die during embryonic development [33]. We tried to delete VCP in SCs and SCPs using conditional *VCP^fl/fl^* mice [34] to determine the role of *Vcp* in PNF initiation and/or maintenance. Unfortunately, the *VCP^fl/fl^;Nf1^fl/fl^;DhhCre* mice died before post-natal day 2. Therefore, genetic deletion of *Vcp* in SCs and SCPs in PNF-bearing mice is not feasible for evaluating its role in PNF development.

### 3.6. Short-Term CB-5083 + PD0325901 Treatment Significantly Inhibits Cell Proliferation and Induces Cell Death

**Figure 5:**
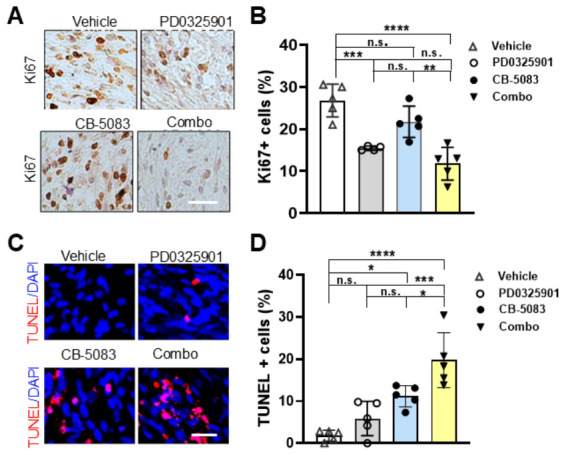
**Short-term CB-5083 + PD0325901 treatment significantly inhibits cell proliferation and induces cell death.** (**A**) Representative images of Ki67 on PNFs removed from *Nf1^fl/fl^;DhhCre* mice that received CB-5083 (80 mg/kg), PD0325901 (1.5 mg/kg), combo, or vehicle control by oral gavage once a day for 5 days (*n* = 5/group). (**B**) Quantification of Ki67+ percentage showing differential inhibitory effects on cells. n.s., no significant difference. (**C**) Representative images of TUNNEL on PNFs removed from *Nf1^fl/fl^;DhhCre* mice that received CB-5083 (80 mg/kg), PD0325901 (1.5 mg/kg), CB-5083 (80 mg/kg) + PD0325901 (1.5 mg/kg) combination, or vehicle control by oral gavage once a day for 5 days. (**D**) Quantification of TUNEL+ percentage showing differential effects on inducing cell death. *n* = 4–5 mice/group. Combo = CB-5083 (80 mg/kg) + PD0325901 (1.5 mg/kg). *, *p* < 0.05; **, *p* < 0.01; ***, *p* < 0.0001, **** *p* < 0.00001. Bar: 20 µM.

Treatment with MEK inhibitors, mirdametinib (PD0325901) and selumetinib, delays PNF growth, but the effects are modest on most of the PNFs and some PNFs re-grow after stopping treatment [6,31]. Based on our in vitro combination results (Figure 3D), we tested if VCPis might provide additional benefit in vivo. We first determined the maximum tolerated dose for CB-5083. Prior reports showed that CB-5083 at 100 mg/kg daily treatment for one week induces protein ubiquitination and cell apoptosis and reduces tumor growth in vivo [35,36]. This dose (in single-reagent or combination treatment) was toxic to *Nf1^fl/fl^;DhhCre* mice after daily treatment for 2 months. After dose de-escalation, we confirmed that the maximum tolerated dose for CB-5083 was 80 mg/kg in this model. All mice survived with less than 20% weight loss in either single-reagent or combination treatment. To evaluate in vivo efficacy, we treated *Nf1^fl/fl^;DhhCre* mice with vehicle control, CB-5083 (80 mg/kg), PD0325901 (1.5 mg/kg), or combination treatment [CB-5083 (80 mg/kg) + PD0325901 (1.5 mg/kg)] for 5 days by oral gavage; the PNFs were removed 2 h after last dose for analysis. Ki67 immunolabeling revealed decreased cell proliferation following either PD0325901 or combination treatment, but not in the VCB-5083-only treatment group (Figure 5A,B). There was no significant difference in cell death between the control and PD0325901 alone; in fact, we have never observed increased cell death following PD0325901 treatment either in vivo or in vitro [31]. Importantly, the combination treatment showed significantly more TUNEL^+^ cells than either CB-5083 or PD0325901 alone (Figure 5C,D).

### 3.7. Long-Term CB-5083 or CB-5083 + PD0325901 Treatment Reduces Tumor Volume, Inhibits Cell Proliferation, and Induces Cell Death/Protein Ubiquitination

**Figure 6:**
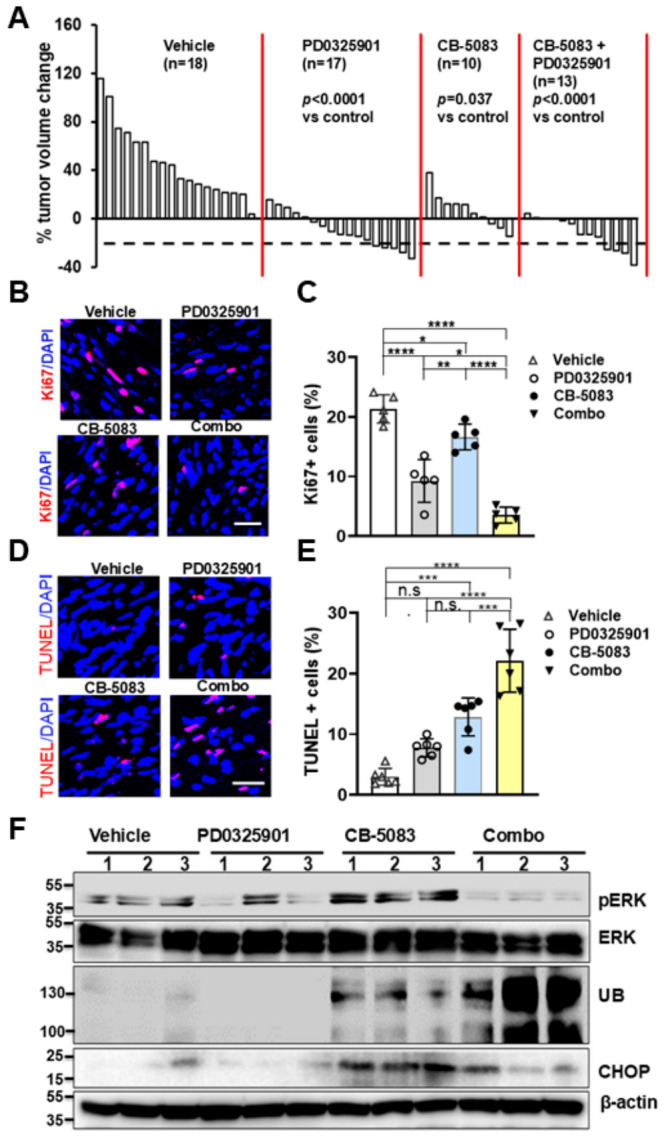
**Long-term CB-5083 or CB-5083 + PD0325901 treatment significantly reduced tumor volume by inhibiting cell proliferation and inducing cell death and ubiquitination.** (**A**) Waterfall plot showing mouse tumor volume change. Data from each mouse are shown as single bars. Change in tumor volume was quantified between 7 and 9 months for each individual mouse treated with vehicle (control) (*n* = 18), PD0325901 (*n* = 17), CB-5083 (*n* = 10), or CB-5083+ PD0325901 (*n* = 13) for 8 weeks. Dashed line indicates a 20% reduction in tumor volume, a threshold used as a surrogate in clinical trials. Red lines separate the treatment groups. (**B**) Representative images of Ki67 on PNFs removed from *Nf1^fl/fl^;DhhCre* mice that received CB-5083 (80 mg/kg), PD0325901 (1.5 mg/kg), CB-5083 (80 mg/kg) + PD0325901 (1.5 mg/kg) combination, or vehicle control treatment by oral gavage once a day for 60 days. (**C**) Quantification of Ki67+ percentage showing differential inhibitory effect on cells (*n* = 5 mice/group). (**D**) Representative images of TUNNEL on PNFs removed from *Nf1^fl/fl^;DhhCre* mice that received CB-5083 (80 mg/kg), PD0325901 (1.5 mg/kg), CB-5083 (80 mg/kg) + PD0325901 (1.5 mg/kg) combination, or vehicle control treatment by oral gavage once a day for 5 days. (**E**) Quantification of TUNEL+ percentage showing differential effect on inducing cell death. n = 6 mice/group. *, *p* < 0.05; **, *p* < 0.01; ***, *p* < 0.0001, **** *p* < 0.00001, n.s., no significant difference. (**F**) Western blot of PNF lysates from 4 different treatments; 3 mice/group. Loading control, β-actin. In (**B**–**E**), combo = CB-5083 (80 mg/kg) + PD0325901 (1.5 mg/kg). Bar: 20 µM.

Finally, we assessed the long-term in vivo effects of inhibiting VCP activity with CB-5083 on PNF growth. We randomized mouse groups and treated the *Nf1^fl/fl^;DhhCre* mice with CB-5083 (80 mg/kg), PD0325901 (1.5 mg/kg), combination treatment, or vehicle control using a five on/two off regimen for 8 weeks. A few mice lost <10% of their weight during the first two weeks but all regained weight thereafter. All mice survived and there were no obvious side effects. As predicted, PD0325901 treatment significantly reduced tumor volume (*p* < 0.0001). Volumetric measurement also showed that the percentage tumor volume changes also significantly decreased in CB-5083- or combination-treated mice compared to vehicle controls (*p* = 0.037 and *p* < 0.0001 respectively) (Figure 6A). However, no difference was observed in the CB-5083 + PD0325901-treated group when compared with CB-5083 (*p* = 0.492) or PD0325901 (*p* = 0.435) monotherapy. CB-5083, PD0325901, and combination treatment all significantly reduced the number of Ki67+ proliferating cells compared to vehicle control, with the CB-5083 + PD0325901 combination showing the greatest effect (Figure 6B,C). TUNEL staining showed that CB-5083 or CB-5083 + PD0325901 treatment significantly increased cell death, while no difference was detected in the PD0325901 treatment group compared to vehicle control (Figure 6D,E). Western blotting revealed unfolding protein response and ER stress response as evidenced by increased CHOP and protein ubiquitination signaling following treatment with the VCPi alone, or in combination with the MEKi. Importantly, the Western blot data also showed that p-ERK was strongly inhibited by the combination treatment (Figure 6F).

## 4. Discussion 

VCP is a highly conserved AAA+ ATPase. It has many functions including protein quality control, ER-associated degradation and ubiquitin–proteasome system maintenance. VCP plays a key role in protein homeostasis and cell survival in normal cells. It is believed that cancer cells can hijack this function to survive cell stress. Thus, VCP is increasingly recognized as a pro-tumorigenic factor and biomarker in many cancers, including non-small cell lung cancer, pancreatic cancer, colorectal cancer, bladder cancer, multiple myeloma, melanoma, breast cancer, hepatocellular carcinoma, and ovarian cancer [37,38]. In this study, we showed that VCP interacts with neurofibromin, the *NF1* gene-encoding protein, in SCs and that its expression is increased in both mouse and human PNFs. The data are consistent with the idea that RAS–MAPK activation in *Nf1^−/−^* cells induces proteostatic stress that increases reliance on VCP to maintain protein homeostasis and cell survival, thereby supporting PNF growth.

VCP interacts with a wide variety of cofactors to regulate proteostasis. More than 40 proteins that bind to VCP’s domains have been identified. These proteins regulate VCP ATPase activity and/or direct VCP to specific locations to carry out functions such as DNA repair, membrane trafficking, or cell cycle control [9]. SVIP, a small regulatory protein, localizes on the ER membrane and acts as a key regulator of VCP-mediated processes, including unfolding protein response and ER-associated degradation. It has been described as having a tumor suppressor role in glioblastoma [39]. We showed that SVIP expression is reduced in human PNFs (Figure 2E) and might act as a negative regulator of VCP to contribute to PNF growth, consistent with a tumor suppressor role. Our results suggest that VCP might be either directly regulated by neurofibromin, or that loss of *Nf1* increases *Runx1* expression, which in turn represses *Svip* expression, leading to activation of *Vcp*. It will be important to define which pathway affects VCP expression in PNFs in the future.

In PNFs, SCPs contribute to tumor formation. We showed that pharmacological and genetic inhibition of VCP decreases mouse neurofibroma sphere number in vitro. Genetic *Vcp* knockdown significantly reduced tumor-like lesion formation in NSG mice (Figure 4E), suggesting that VCP itself, or in coordination with RAS-MAPK signaling, contributes to PNF initiation.

VCP contributes to tumor growth, suggesting that targeting VCP might be a good therapeutic approach. VCP uses energy from ATP hydrolysis to unfold and segregate ubiquitinated protein substrates for degradation. When VCP’s ATPase activity is inhibited, ubiquitinated proteins accumulate, leading to cell stress and often initiating programmed cell death (apoptosis). CB-5083, a potent VCPi with moderate oral bioavailability, was identified in 2015. CB-5083 binds to the D2 domain of VCP to inhibit VCP ATPase activity and leads to retention of ER-associated degradation and generation of irresolvable proteotoxic stress, therefore inducing apoptosis. It inhibits tumor growth in hematological xenograft models [35,40]. CB-5083 suppresses osteosarcoma growth and stem cell properties by altering protein homeostasis [41]. Zhang et al. show that pharmacological inhibition of VCP by CB-5083 partially abolished the oncogenic effects of TMEM63A on triple-negative breast cancer progression both in vitro and in vivo [42]. Single-agent CB-5083 treatment inhibited xenografted bladder cancer growth and CB-5083 effectively killed tumor cells and acted as a radio-sensitizing agent both in vitro and in vivo [43] in multiple myeloma [44]. Targeting VCP enhanced colorectal cancer therapy through STING stabilization [45]. The differential inhibitory effects between WT SCs and PNF SCs on cell growth (Figure 3A) and protein ubiquitination (Figure 3B) indicated potential therapeutic efficacy in PNFs by targeting VCP. We showed that inhibition of VCP significantly reduced PNF growth compared to control (Figure 6A), suggesting that VCP might be an alternative therapeutic target.

In vitro CB-5083 + PD0325901 combination treatment resulted in protein ubiquitination and p-ERK inhibition, while single-reagent treatment only led to protein ubiquitination (CB-5083) or p-ERK inhibition (PD0325901) (Figure 3D), suggesting that co-targeting VCP and MEK might provide synthetic lethality. Importantly, we detected increased cell death in both the 5-day and 60-day combination treatment groups. Although combination showed a trend of relative better efficacy, we did not detect a significant difference in reducing PNF volume percentage change compared to single-reagent PD0325901 or CB-5083 treatment (Figure 6A). It is possible that: (1) PD0325901 or CB-5083 each has very strong effects on reducing tumor volume, so we did not have enough power (i.e., mouse number) to reach statistical significance; (2) VCP functions in parallel to, or is modulated by, RAS–MAPK signaling under stress or oncogenic conditions; (3) elevated VCP expression is a secondary adaptive response to RAS-MAPK-driven stress rather than a direct signaling target; (4) other unidentified pathway(s) might be involved or compensated in PNF initiation/maintenance upon inhibition of VCP and MEK; (5) the combination did not kill SCPs, the PNF-initiating cells. Future work should focus on identifying the specific cell populations that die in response to CB-5083 or combination treatment.

The first generation VCPi (CB-5083) tested here showed some off-target effects: two phase I clinical trials (NCT02243917 and NCT02223598) were terminated due to adverse effects on vision resulting from inhibition of phosphodiesterase-6 [46]. In 2021, a second-generation VCPi, CB-5339, was developed and it showed efficacy in several multiple-AML models [47]. This compound has progressed through early clinical evaluation with the successful completion of a phase I study in acute myeloid leukemia, or myelodysplastic syndrome (NCT04402541), providing important data on safety, tolerability, and pharmacokinetics. A second phase I study on testing the safety of CB-5339 in patients with solid tumors that have spread to other places in the body (advanced) or lymphomas (NCT04372641) was withdrawn for unreported reasons. Overall, these early clinical experiences have informed dose selection and study design, supporting the continued advancement of this inhibitor. It will be interesting to test CB-5339 in our PNF mouse model and compare its effect on PNF growth with that of CB-5083 in the future.

## 5. Conclusions

**Figure 7:**
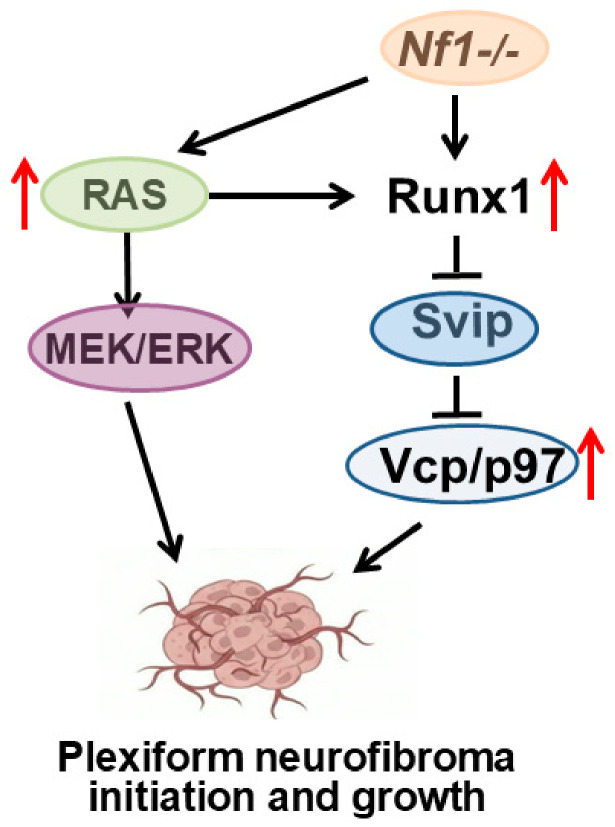
**A schematic model of plexiform neurofibroma initiation and growth.** Loss of *Nf1* increases RAS activity, thereby activating the MEK/ERK signaling pathway and contributing to PNF formation. In the meantime, loss of *Nf1* upregulates *Runx1* expression, which binds to SVIP to enhance VCP expression, thereby further contributing to PNF pathogenesis.

It is well established that loss of *Nf1* increases RAS activity, thereby activating the MEK/ERK signaling pathway and contributing to PNF formation. We demonstrate that VCP interacts with neurofibromin. Furthermore, loss of *Nf1* upregulates *Runx1* expression, which binds to SVIP to enhance VCP expression, thereby further contributing to PNF pathogenesis (Figure 7). VCP expression is increased in both mouse and human PNFs. It might be regulated by and functionally coupled to RAS–MAPK-driven SC states. The significant effects of VCP inhibition in this pre-clinical study suggest a potential novel therapy for patients with PNFs.

## Data Availability

The original contributions presented in this study are included in the article. Further inquiries can be directed to the corresponding author.
